# Socioeconomic disparity in cervical cancer screening among Korean women: 1998–2010

**DOI:** 10.1186/1471-2458-13-553

**Published:** 2013-06-06

**Authors:** Minjee Lee, Eun-Cheol Park, Hoo-Sun Chang, Jeoung A Kwon, Ki Bong Yoo, Tae Hyun Kim

**Affiliations:** 1 Department of Public Health, Institute of Health Services Research, Yonsei University, Seoul, Korea; 2 Department of Preventive Medicine and Public Health, Institute of Health Services Research, College of Medicine, Yonsei University, Seoul, Korea; 3 Graduate School of Public Health, Institute of Health Services Research, College of Medicine, Yonsei University, Seoul, Korea

**Keywords:** Cervical cancer, Screening, Socioeconomic status, Disparity

## Abstract

**Background:**

Cervical cancer is the sixth most common cause of cancer among Korean women and is one of the most preventable cancers in the world. This study aimed to investigate the change in cervical cancer screening rates, the level of socioeconomic disparities in cervical cancer screening participation, and whether there was a reduction in these disparities between 1998 and 2010.

**Methods:**

Using the Korean Health and Nutrition Examination Survey, women 30 years or older without a history of cervical cancer and who completed a health questionnaire, physical examination, and nutritional survey were included (n = 17,105). Information about participation in cervical cancer screening was collected using a self-administered questionnaire. Multiple logistic regression analysis was performed to investigate the relationship between cervical cancer screening participation and the socioeconomic status of the women.

**Results:**

The cervical cancer screening rate increased from 40.5% in 1998 to 52.5% in 2010. Socioeconomic disparities influenced participation, and women with lower educational levels and lower household income were less likely to be screened. Compared with the lowest educational level, the adjusted odds ratios (ORs) for screening in women with the highest educational level were 1.56 (95% confidence interval (CI): 1.05–2.30) in 1998, and 1.44 (95% CI: 1.12–1.87) in 2010. Compared with women with the lowest household income level, the adjusted ORs for screening in women with the highest household income level were 1.80 (95% CI: 1.22–2.68), 2.82 (95% CI: 2.01–3.96), and 1.45 (95% CI: 1.08–1.94) in 2001, 2005, and 2010, respectively.

**Conclusion:**

Although population-wide progress has been made in participation in cervical cancer screening over the 12-year period, socioeconomic status remained an important factor in reducing compliance with cancer screening.

## Background

Cervical cancer is one of the most preventable cancers in the world but it is the eighth leading cause of cancer-related deaths in Korea [1,2]. Regular Papanicolaou (Pap) tests are an excellent diagnostic tool for detecting not only cancerous, but also precancerous cells, both of which can be removed [3-5]. Previous observational studies have consistently shown dramatic reductions in the cervical cancer mortality rate after the implementation of population-based screening programs [6,7].

Since its introduction in the 1940s, the Pap smear has been associated with sharp declines in cervical cancer incidence and mortality [8,9]. In Korea, the age-adjusted incidence of cervical cancer dropped from 18.6 (per 100,000) in 1999 to 12.0 in 2009, and its mortality declined from6.2 per 100,000 in 1995 to 3.8 per 100,000 in 2009 [1,10].

In Korea, there are currently three main cancer screening programs [10], namely the National Cancer Screening Program (NCSP), the Korea National Health Insurance (NHI) program, and screening services voluntarily provided by independent medical facilities. In 1999, the Korean government created the NCSP and established a 10-year plan for cancer control [11]. The NCSP provided free cancer screening services for stomach, breast, and cervical cancers to medical aid recipients between 1999 and 2001 [12]. In 2002, coverage of free cancer screening was expanded to NHI beneficiaries within the lowest 20% income bracket, and in 2003, those within the lowest 30% income bracket were included in the target population. From 2005, the NCSP expanded coverage of free screening for stomach, breast, cervical, liver and colorectal cancer to Medical Aid recipients, and the NHI included beneficiaries who were within the lower 50% of income earners [13].

Despite these public health efforts, the rate of cervical cancer screening may not be uniform across groups with different socioeconomic status. Previous studies suggested that socioeconomic disparities existed in cancer screening rates [14,15], and, in particular, global evidence suggested that the cervical cancer screening rate was influenced by socioeconomic factors as well as demographic factors such as race [16-21]. Studies in the United States and Korea also showed that socioeconomic disparities continued in cervical cancer screening participation, though there has been an improvement in overall screening rate [14,22].

Although the above-mentioned studies are informative in identifying important factors influencing cervical cancer screening, they are either cross-sectional studies or not nationally representative, or their study periods were in the late 1990s or the early 2000s. To achieve timely and challenging objectives in public health, such as improvement in cancer screening rates with a reduction in socioeconomic disparities, it is necessary to monitor the long-term trend. Therefore, the objective of this study was to investigate the changes in cervical cancer screening rate over the 12-year period from 1998 to 2010 in a nationally representative sample of Korean women, and to examine whether socioeconomic disparities in cervical cancer screening rates have been reduced over this period.

## Methods

### Data source and subjects

This study used data from the 1998–2010 Korea National Health and Nutrition Examination Survey (KNHANES). The KNHANES is a nationally-representative study managed by the Korean Ministry of Health and Welfare. Participants were enrolled from the household registry using a stratified multistage probability design. The KNHANES consists of four parts: a health interview survey, a health behavior survey, a physical examination, and a nutritional survey. Trained interviewers conducted all surveys and trained healthcare professionals conducted all physical examinations. All participants provided informed consent before participation in the KNHANES.

There were 211,116 women aged 30 years or older who completely answered the health behavior survey between 1998 and 2010. Women who did not provide information about cervical cancer screening or nutrition or who did not have an additional physical examination were excluded from the study. Finally, a total of 17,105 women (2,725 in 1998, 1,622 in 2001, 2,596 in 2005, 2,944 in 2008, and 2,737 in 2010) were included in the analysis.

### Independent variables and outcome variables

From 1998 to 2001, participants were asked, “*Have you ever been screened for cervical cancer?*” and answers were recorded as either yes or no. From 2005 to 2010, participants were asked, “*When was the last time you were screened for cervical cancer?*” and answers were recorded as either never, less than 1 year ago, 1–2 years ago, or more than 2 years ago. According to the Korean NCSP guidelines, women 30 years of age and older should receive a Pap smear test every 2 years. In the present study, the outcome variable was whether participants adhered to the Korean NCSP guidelines. We defined participants as not adhering to the NCSP guidelines if they reported never being screened for cervical cancer or were examined more than 2 years prior to completing the questionnaire.

Based on a literature review, we chose several variables as possible factors related to screening participation. Thus, our primary variables of interest were socioeconomic factors, including education, household income, and occupation. Other variables included in the study were age, marital status, health insurance type, health status (limitation in general activities and perceived health status), and health behavior (smoking and obesity). Educational status was divided into three groups: none or elementary school, middle school to high school, and university or higher. Household income, provided by the KNHANES, was calculated by dividing the monthly household income by the square root of the household size, and grouped into four household income quartiles. Occupation was categorized as “white collar (manager, professional level, office workers, service workers, sales)”, “blue collar (agriculture, fishery, technicians, mechanics, assemblers, simple labor)”, and “others (student, housewife, unemployed)”. Marital status was “married” vs. “not married”. Health insurance type was categorized as national health insurance for the self-employed, national health insurance for those not self-employed, and being in receipt of Medical Aid. Health status and health behavioral factors included limitation in general activities (yes, no), perceived health status (good or regular vs. bad), smoking (non, ex or current), and body mass index (BMI), categorized as < 18.5, 18.5– < 23, 23– < 25, and ≥ 25 kg/m^2^ according to the guidelines provided by the World Health Organization West Pacific Region (2000).

### Statistical analysis

The KNHANES was based on a complex sample design. Therefore, all statistical analyses were performed using the survey procedure of SAS version 9.2 (SAS Inc., Cary, NC, USA), specifically designed to analyze such sample survey results. In the survey procedure, information pertaining to complex sample designs such as stratification, clustering, and unequal weighting is combined to analyze the parameters.

We used descriptive statistics for the characteristics of the subjects, and reported the number and percentage for each variable. The participation rates in cervical screening were calculated according to all variables. The odds ratios (ORs) and 95% confidence intervals (CIs) were calculated to measure the strength of the association between the measured variables and screening participation. We regarded a *p*-value of less than 0.05 as statistically significant.

## Results

### Baseline characteristics and participation in cervical cancer screening

The characteristics of the study population and participation rate in cervical cancer screening from 1998 to 2010 are summarized in Table 1. In this study, the majority of women were married, and enrolled in the NHI program. Most women reported no limitation in their daily activities and were non-smokers. The cervical cancer participation rates increased from 40.5% in 1998 to 52.5% in 2010.

**Table 1:** Basic characteristics of the study population and participation rate in cervical cancer screening in women ≥30 years, 1998–2010

**Variables**	**1998**					**2001**					**2005**					**2008**					**2010**				
	**Total**	**%**	**Screen**	**%**	**p-value**	**Total**	**%**	**Screen**	**%**	**p-value**	**Total**	**%**	**Screen**	**%**	**p-value**	**Total**	**%**	**Screen**	**%**	**p-value**	**Total**	**%**	**Screen**	**%**	**p-value**
**Age (years)**																									
30-39	715	30.1	362	50.9	<.0001	369	23.6	191	50.6	<.0001	710	29.7	362	51.3	<.0001	707	26.4	363	51.8	<.0001	617	24.1	317	51.9	<.0001
40-49	641	24.9	358	55.9		373	23.8	217	58.8		709	27.7	421	59.0		663	28.1	393	60.2		556	26.6	340	61.1	
50-59	557	18.5	219	39.0		310	20.1	132	43.9		469	17.5	217	44.1		562	20.6	312	54.3		623	22.3	385	60.8	
60-69	497	15.6	101	21.6		315	18.3	79	26.3		397	13.1	123	34.2		536	13.4	208	40.7		502	13.6	250	49.1	
70+	315	11.0	25	6.4		255	14.1	27	9.1		311	11.9	43	14.7		476	11.5	86	17.9		439	13.4	116	26.3	
**Education**																									
None or elementary school	1,414	45.5	381	26.1	<.0001	751	44.3	186	25.4	<.0001	897	32.1	260	29.9	<.0001	1,138	29.9	375	35.5	<.0001	1,294	43.8	563	43.6	<.0001
Middle or high school	1,089	44.6	564	52.0		688	44.2	360	53.1		1,224	48.8	641	52.1		1,239	48.6	643	51.9		1,023	40.9	588	59.4	
University or higher	222	10.0	120	54.4		183	11.6	100	53.4		475	19.1	265	55.2		567	21.5	344	62.6		420	15.3	257	59.5	
**Marital status**																									
Married	2,073	77.2	944	46.8	<.0001	1,181	73.0	539	45.8	<.0001	1,984	76.0	999	50.6	<.0001	664	18.6	199	32.0	<.0001	2,202	81.3	1,202	55.2	<.0001
not married	652	22.8	121	19.1		441	27.0	107	27.6		612	24.0	167	29.6		2,280	81.4	1,163	53.2		535	18.7	206	40.8	
**Household income**																									
Quartile 1	770	24.6	184	24.8	<.0001	509	30.4	109	23.8	<.0001	666	23.0	187	27.6	<.0001	694	17.0	220	33.8	<.0001	635	20.6	252	40.7	<.0001
Quartile 2	643	22.0	247	39.6		407	23.8	175	44.0		624	25.6	255	40.8		756	27.3	328	47.5		685	27.1	336	51.5	
Quartile 3	708	27.6	345	49.0		347	22.4	170	49.1		669	26.5	343	51.2		759	28.1	382	50.9		710	27.3	391	55.0	
Quartile 4	604	25.8	289	47.0		359	23.5	192	52.1		637	24.9	381	61.0		735	27.6	432	58.9		707	25.0	429	60.6	
**Health insurance type**																									
NHI (self-employed)	1,482	53.7	597	41.3	<.0001	748	46.2	296	40.0	0.002	1,066	42.5	451	44.0	0.064	1,156	39.9	524	48.4	0.001	932	36.7	446	49.8	0.252
NHI (employee)	1,120	42.3	453	42.3		780	48.1	333	44.0		1,412	53.5	671	47.5		1,662	56.9	805	51.1		1,712	59.6	919	54.0	
Medical Aid	123	4.0	15	9.6		94	5.7	17	22.1		118	4.0	44	35.5		126	3.2	33	28.0		93	3.8	43	56.1	
**Occupation**																									
white collar	481	20.3	236	48.9	<.0001	312	19.4	165	50.5	0.002	663	25.8	352	52.7	0.003	628	23.9	344	54.0	0.024	670	27.7	369	55.0	0.233
blue collar	836	23.8	290	35.4		324	20.1	124	40.9		545	18.8	232	43.6		751	21.4	315	45.3		569	21.4	282	54.0	
others	1,408	55.9	539	39.6		986	60.6	357	37.8		1,388	55.4	582	42.9		1,562	54.7	700	48.7		1,498	50.8	757	50.5	
**Limitation in general activities**																									
Yes	709	27.4	293	44.5	0.013	201	12.8	62	33.7	0.045	349	12.5	104	30.8	<.0001	743	21.4	279	44.1	0.019	929	31.3	433	49.1	0.024
No	2,016	72.6	772	38.9		1,421	87.2	584	41.9		2,247	87.5	1,062	47.7		2,201	78.6	1,083	50.7		1,808	68.7	975	54.1	
**Perceived health status**																									
Good or regular	1,675	63.9	699	43.0	0.001	1,014	63.3	456	45.5	<.0001	1,807	70.7	891	49.8	<.0001	1,993	71.9	956	50.2	0.165	2,029	74.8	1,093	54.1	0.016
Bad	1,050	36.1	366	36.0		608	36.7	190	32.9		789	29.3	275	35.3		951	28.1	406	46.8		708	25.2	315	47.8	
**Smoking**																									
Non-smoker	2,445	89.7	1,004	42.2	<.0001	1,499	92.1	618	42.4	0.001	2,375	90.8	1,101	46.9	0.000	2,644	88.7	1,248	49.9	0.094	2,503	90.1	1,299	52.7	0.026
Ex-smoker	72	3.0	15	23.4		24	1.6	4	17.7		104	4.3	38	53.9		148	5.5	61	49.7		133	5.4	72	60.1	
Current smoker	208	7.2	46	25.4		99	6.2	24	25.0		117	4.9	27	28.4		152	5.8	53	38.8		101	4.5	37	40.7	
**Body mass index (kg/m**^**2**^**)**																									
<18.5	106	3.8	24	26.8	0.007	65	3.7	21	31.0	0.165	81	3.0	35	42.2	0.066	123	4.3	50	40.9	0.030	101	3.7	45	45.5	0.000
18.5 ≤ 23	1,076	40.4	437	43.1		601	37.9	257	43.4		4,050	41.3	493	48.0		1,209	43.1	580	52.3		1,150	42.5	624	55.8	
23 ≤ 25	637	23.6	262	41.8		399	24.4	160	42.3		637	23.7	292	47.4		683	23.1	320	50.4		640	23.5	358	56.6	
25≤	906	32.2	342	37.7		557	33.9	208	38.2		828	31.9	346	41.3		929	29.5	412	45.2		846	30.3	381	45.6	
**total**	2,725	100.0	1,065	40.5		1,622	100.0	646	40.9		2,596	100.0	1,166	45.5		2,944	100.0	1,362	49.3		2,737	100.0	1,408	52.5	

*NHI*, National health insurance.

Women with the lowest educational status had a participation rate of 26.1% in 1998 and 43.6% in 2010. However, women with the highest educational status reported a higher participation rate of 54.4% in 1998 and 59.5% in 2010. Women in the lowest household income group had a participation rate of 24.8% in 1998 and 40.7% in 2010. Women in the highest household income group had a participation rate of 47.0% in 1998 and 60.6% in 2010.

**Figure 1:**
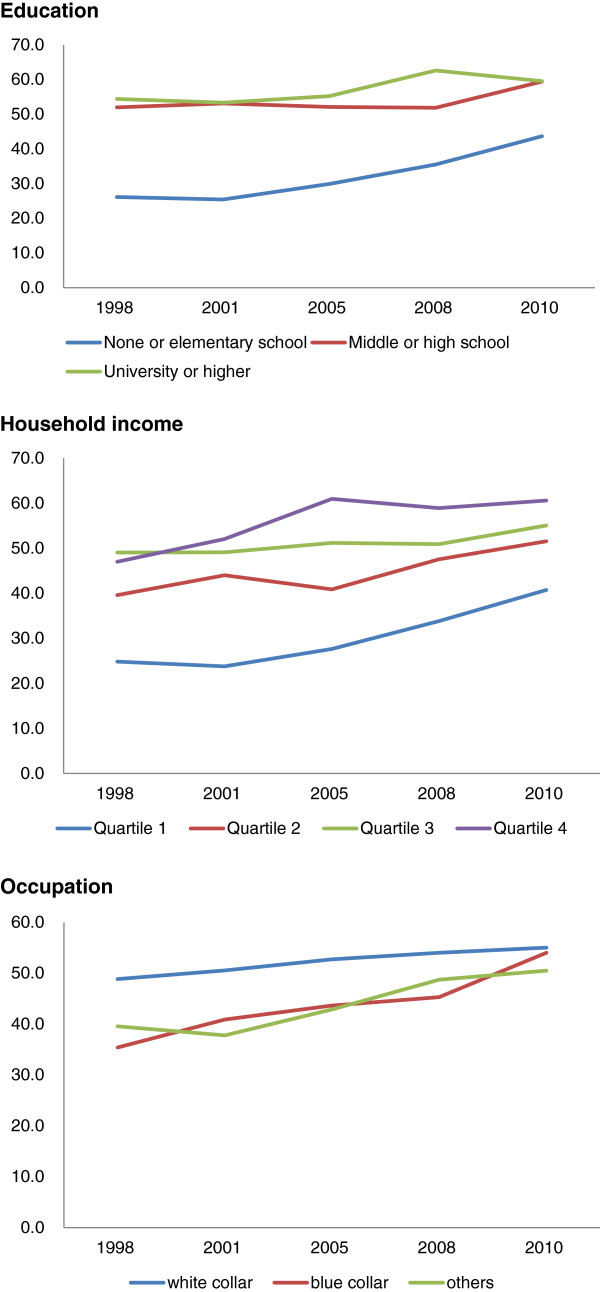
Cervical cancer screening rate by education and household income and occupation, 1998–2010.

Figure 1 indicates that the gaps between the highest and lowest educational status and income groups narrowed during the 12 years in Korea.

### Factors associated with cervical cancer screening participation

Table 2 shows the results of the multivariate logistic regression analysis for cancer screening. Of the socioeconomic factors considered, higher educational level was found to be associated with a higher OR in 1998, 2001, 2008, and 2010. Compared with the lowest educational level, the adjusted ORs of the highest education level were 1.56 (95% CI: 1.05–2.30), 1.90 (95% CI: 1.26–2.87), and 1.73 (95% CI: 1.12–2.66) in 1998, 2008, and 2010. A higher household income was also found to be associated with a higher OR in 2001, 2005, and 2010. Compared with the lowest household income level, the adjusted ORs of the highest household income level were 1.80 (95% CI: 1.22–2.68), 2.82 (95% CI: 2.01–3.96), and 1.45 (95% CI: 1.08–1.94) in 2001, 2005, and 2010, respectively.

**Table 2:** Factors associated with cervical cancer screening among women ≥30 years, 1998–2010

**Variables**	**1998**		**2001**		**2005**		**2008**		**2010**	
**Education**										
None or elementary school	1.00		1.00		1.00		1.00		1.00	
Middle or high school	1.43	(1.13-1.82)	1.67	(1.18-2.38)	1.15	(0.82-1.61)	1.24	(0.93-1.66)	1.71	(1.24-2.35)
University or higher	1.56	(1.05-2.30)	1.56	(0.94-2.61)	1.00	(0.64-1.56)	1.90	(1.26-2.87)	1.73	(1.12-2.66)
**Household income**										
Quartile 1	1.00		1.00		1.00		1.00		1.00	
Quartile 2	1.22	(0.92-1.62)	1.65	(1.16-2.34)	1.39	(1.00-1.93)	1.09	(0.82-1.46)	1.12	(0.84-1.49)
Quartile 3	1.52	(1.11-2.07)	1.72	(1.15-2.59)	1.93	(1.44-2.59)	1.05	(0.78-1.40)	1.21	(0.89-1.66)
Quartile 4	1.31	(0.95-1.81)	1.80	(1.22-2.68)	2.82	(2.01-3.96)	1.34	(0.97-1.84)	1.45	(1.08-1.94)
**Occupation**										
white collar	1.00		1.00		1.00		1.00		1.00	
blue collar	0.92	(0.69-1.23)	1.08	(0.70-1.66)	1.01	(0.72-1.41)	1.01	(0.78-1.32)	1.29	(0.95-1.76)
others	1.12	(0.87-1.44)	1.02	(0.79-1.35)	1.04	(0.79-1.35)	1.18	(0.93-1.49)	1.20	(0.92-1.57)

Results are presented as adjusted odds ratios and (95% confidence intervals), and adjusted for age, marital status, health insurance type, limitation in general activities, perceived health status, smoking, and body mass index.

Among the other variables, age was a statistically significant factor which was inversely related to cervical cancer screening during 1998–2010, suggesting that older women were less likely to participate in screening. Although marital status, health insurance type, and smoking status were statistically significant factors in one or two study years, their significance was either not as strong as socioeconomic status or somewhat inconsistent.

## Discussion

The objective of this study was to examine the change in rates of participation in cervical cancer screening among Korean women from 1998 to 2010, and to test whether socioeconomic disparities in cervical cancer screening decreased, stayed the same, or worsened. We observed that the participation rate of Korean women 30 years or older in cervical cancer screening was 40.5% in 1998, 40.9% in 2001, 45.5% in 2005, 49.3% in 2008, and 52.5% in 2010. Although this suggests that there has been steady progress in improving the cervical cancer screening rate over the past decade, there is certainly room for improvement because the rate is still around 50%, significantly lower than in other economically developed countries. There were particularly low rates of participation in women with the lowest educational level (26.1% in 1998, 25.4% in 2001, 29.9% in 2005, 35.5% in 2008, and 43.6% in 2010), and in women with the lowest household income (24.8% in 1998, 23.8% in 2001, 27.6% in 2005, 33.8% in 2008, and 40.7% in 2010). Importantly, the participation rates of women in the lowest education and income groups markedly improved over the years, and the gaps with the highest education and income groups were reduced. The results of our study suggest important policy implications for policymakers to improve participation rates and to further reduce the difference in rates according to socioeconomic status.

Previous studies have found educational level to be a significant predictor of cervical cancer screening participation [23,24], and educational level has a huge effect on knowledge of the advantages of participation in cervical cancer screening after controlling for other covariates [25,26]. The results of our study are consistent with previous studies in showing that educational level was significantly associated with participation in cervical cancer screening among Korean women, and, more importantly, that the association lasted over a decade. It is worth noting that two previous studies found that disparities in cancer screening by household income were improved, but there was no improvement for disparities in cancer screening by education level among Korean women [27,28].

Previous studies also found that household income was a significant predictor of cervical cancer screening participation [28,29]. It was suggested that to improve cancer screening participation rates in lower income individuals, a primary health care intervention such as an organized program of cervical screening that focuses on deprived groups is needed [30]. Therefore, it is important to keep monitoring how public health policies impact on participation rates over time, such as that which expanded the scope of free cervical cancer examinations to women in the lower 50% income bracket of households [13].

Our study has several limitations. First, although this study examined data in a 12-year study period, it was based on pooled cross-sectional data, from which we cannot detect a causal relationship. Second, the KNHANES is based on self-reported responses to participation in cervical cancer screening, which may raise acquiescence bias or recall bias. To minimize recall bias in collecting the data, the KNHANES was conducted by educated and trained interviewers. However, we acknowledge that the survey was unable to perform a cross-check with medical records. Therefore, recall and acquiescence (social desirability) bias can remain, and may result in misclassification. Although misclassification can be either random or nonrandom, we believe that, in a large nationwide survey such as KNHANES, it was random. Therefore, potential recall bias may lead to an association toward null, and an underestimate of the true association. A previous study also pointed out a similar possibility of underestimation of the actual participation rate [31-33]. Finally, other factors that may be significant determinants of cervical cancer screening participation were not included in the current study. For example, there was no control for family history of cervical cancer, age at first sexual intercourse, and knowledge and attitudes about cervical cancer risk factors and benefits of the Pap test.

## Conclusion

In conclusion, in the analysis of nationally representative data over a decade, we found that there was an increase in participation in cervical cancer screening programs by Korean women from 40.5% in 1998 to 52.5% in 2010, though the rate remained lower than in other developed countries. We also observed that despite the overall increase in screening rates, socioeconomic disparities continued to exist. Although screening rates in women with the lowest educational levels and household incomes improved over the period, they remained lower than in women of the highest education and income groups.

These results demonstrate the need for more aggressive interventions and policies to improve participation in cervical cancer screening especially for those at a lower income and education level. Analyses of cervical cancer screening rates by measures of household income, educational level, and other factors over the long term may help policy-makers to better direct their resources to those of greatest need. Ensuring that free cervical cancer screening programs or other public health programs remain available for women in the lower income groups can lead us closer to national screening goals, yet policies or campaigns still need to address disparities in cervical cancer screening according to educational level.

## Competing interests

The authors declared that they have no competing interest.

## Authors’ contributions

All the authors developed the idea for the study. ML analyzed the data and wrote the first draft of the paper. ECP and HSJ participated in the design and coordination. JAK and KBY contributed to interpreting the data. THK contributed to developing and writing subsequent drafts. All authors approved the final manuscript. THK is the guarantor.

## Pre-publication history

The pre-publication history for this paper can be accessed here:

http://www.biomedcentral.com/1471-2458/13/553/prepub
